# Insomnia Interventions in the Workplace: A Systematic Review and Meta-Analysis

**DOI:** 10.3390/ijerph17176401

**Published:** 2020-09-02

**Authors:** Juan Vega-Escaño, Ana María Porcel-Gálvez, Rocío de Diego-Cordero, José Manuel Romero-Sánchez, Manuel Romero-Saldaña, Sergio Barrientos-Trigo

**Affiliations:** 1 Spanish Red Cross Nursing School, University of Seville, Avda. Cruz Roja 1, 41009 Seville, Spain; juanvegadue@gmail.com; 2 Department of Nursing, University of Seville, 41009 Seville, Spain; aporcel@us.es (A.M.P.-G.); sbarrientos@us.es (S.B.-T.); 3 Faculty of Nursing and Physiotherapy, University of Cádiz, 11009 Cádiz, Spain; jose.romerosanchez@mail.uca.es; 4 Department of Nursing, University of Córdoba, 14001 Córdoba, Spain; z92rosam@uco.es

**Keywords:** insomnia, occupational health, meta-analysis, workplace health, systematic review

## Abstract

The aim of this systematic review and meta-analysis was to identify and evaluate the impact of interventions to improve or reduce insomnia in the workforce through randomized clinical trials. Following the recommendations of the PRISMA and MARS statement, a systematic literature search was carried out on the PubMed, Web of Science, CINHAL, and PsycINFO databases, with no restrictions on the language or publication date. For the meta-analysis, a random-effects model and the *Insomnia Severity Index* were used as outcome measures. To assess the risk of bias and the quality of evidence, the Cochrane Collaboration tool and the GRADE method were used, respectively. Twenty-two studies were included in the systematic review and 12 studies in the meta-analysis, making a total of 14 intervention groups with a sample of 827 workers. Cognitive behavioral therapy was the most widely used intervention. According to the estimated difference between the means, a moderate effect for the reduction of insomnia symptoms after the intervention (MD −2.08, CI 95%: [−2.68, −1.47]) and a non-significant degree of heterogeneity were obtained (*p* = 0.64; I^2^ = 0%). The quality of the evidence and the risk of bias were moderate. The results suggest that interventions on insomnia in the workplace are effective for improving workers’ health, and that improvements in the quality of sleep and a decrease in the symptoms of insomnia are produced, thanks to an increase in weekly sleeping hours and a reduction in latency at sleep onset. As regards work, they also led to improvements in productivity, presenteeism, and job burnout.

## 1. Introduction

Insomnia is the most common sleep disorder worldwide [1] and was defined in the fifth edition of the Diagnostic and Statistical Manual of Mental Disorders as a dissatisfaction with sleep owing to difficulties in falling asleep, staying asleep, or waking up too early [2]. However, there is a lack of consensus over its diagnosis due to differences in sensitivity and specificity between the different methods used to identify it (actigraphy, polysomnography, and self-reports, among others). As a consequence, its prevalence is extremely heterogeneous, between 5.7% and 55.8%, and it is more common among women and people with a history of psychiatric illness or a low socio-economic status [3,4,5].

In addition, insomnia is one of the most prevalent occupational risks among the working population [6,7], being directly responsible for multiple impacts on workers’ health on both a physical and mental level [8]. Moreover, it has been proven that in the workplace, there is an association between insomnia and occupational accidents, productivity, presenteeism, sick leave, and work-related burnout [9,10,11,12]. 

There are various observational studies that currently describe the different causes and consequences of work-related insomnia. In a cross-sectional study among Danish employers, it was shown that sedentary and physical workers with sleep problems performed less high-intensity leisure time physical activity, after adjustment for various potential confounders (age, gender, lifestyle factors) and factors related to work, work group, and chronic diseases [13]. Other researchers have linked metabolic syndrome (SME) with sleep problems and have demonstrated that low quantity and quality of sleep are powerful predictors of SME [14]. Furthermore, Hu et al., (2019) conducted a study in 472 Taiwanese flight attendants to learn how physical, mental, and work-related factors affected their ability to work. Their findings indicated that 43.6% had insomnia, which was the most negative impact on these attendants’ working capacity.

Previously, various meta-analyses had studied the efficiency of interventions for the treatment of insomnia from different approaches, looking at the effectiveness of either cognitive behavioral therapy or interventions for mental disorders aimed at improving various factors, including insomnia. Researchers evaluating e-mental health interventions for every mental health condition in an occupational context found statistically significant moderate effects for insomnia (Hedges’g = 0.70), compared with control groups with waitlist [15]. Another study carried out by German researchers on an internet-based cognitive-behavioral therapy for insomnia among employees showed positive effects for the improvement of the quality of sleep, which leads to a reduction in the insomnia symptoms [16]. 

Reviews of the literature reveal strong evidence of significant associations between insomnia and employees’ health, although gaps in the research were identified in the quantitative synthesis of these aspects. Further gaps were also identified in previous meta-analyses: some studies focused on reviewing the efficacy of mental health interventions to address insomnia (here, it should be noted that occupational interventions addressing areas of mental health can lead to treatments being less effective due to problems in the estimation of mental disorders), while others examined the therapeutic effects of *Cognitive Behavioral Therapy for Insomnia* (CBT-I) only in the non-working population.

Therefore, the aim of this study was to identify and evaluate the impact of interventions for improving or reducing insomnia in the workforce through randomized clinical trials (RCT).

## 2. Methodology

### 2.1. Protocol

This study was carried out in accordance with the systematic reviews and meta-analyses protocols guidelines of the PRISMA statement [17] and the Meta-Analysis Reporting Standards (MARS) of the American Psychological Association (APA) [18]. Before beginning the study, two external reviewers evaluated the protocol separately. The protocol of the review available for consultation was recorded in the PROSPERO database (CRD42020149383 http://www.crd.york.ac.uk).

### 2.2. Databases and Search Strategy

A systematic search was carried out on 12 December 2018 by two separate authors (J.V.E. and R.D.C.) on the following databases: SCOPUS, PubMed, Web of Science, CINHAL, and PsycINFO with no restrictions on the language of publication and with a publication date between 1961 and 2019. The keywords used were obtained from the Medical Subject Headings (MeSH) and were merged together in Boolean expressions for each database searched (Supplementary Materials Table S1). A third researcher (J.M.R.S.) reviewed the citations of the systematic reviews and the meta-analysis obtained through the searches on the databases. The search was updated on 29 December, 2019 to retrieve potential records published since the review start date.

### 2.3. Eligibility Criteria

The criteria of participant, intervention, comparison, result, time, and study design (PICOTS) were used to establish the eligibility criteria of the study. The citations were entered into the Mendeley reference management software (version 1.19.4).

- Participants: studies carried out on currently employed workers were considered.
- Intervention/exposure: studies that evaluated interventions aimed at reducing insomnia in workers were considered, either as a primary or secondary outcome measure.
- Comparison: we also considered studies that compared the results obtained after the intervention on insomnia in workers with results from those workers who, after being recruited from the same population group, did not undergo any intervention, and stayed on the waiting list or continued with their usual activities.
- Results: any study obtaining primary or secondary results on work-related insomnia were considered.
- Time: original studies with no restrictions on the date of publication were included.
- Study design: only randomized controlled trials (RCT) were included.

### 2.4. Study Selection

**Figure 1:**
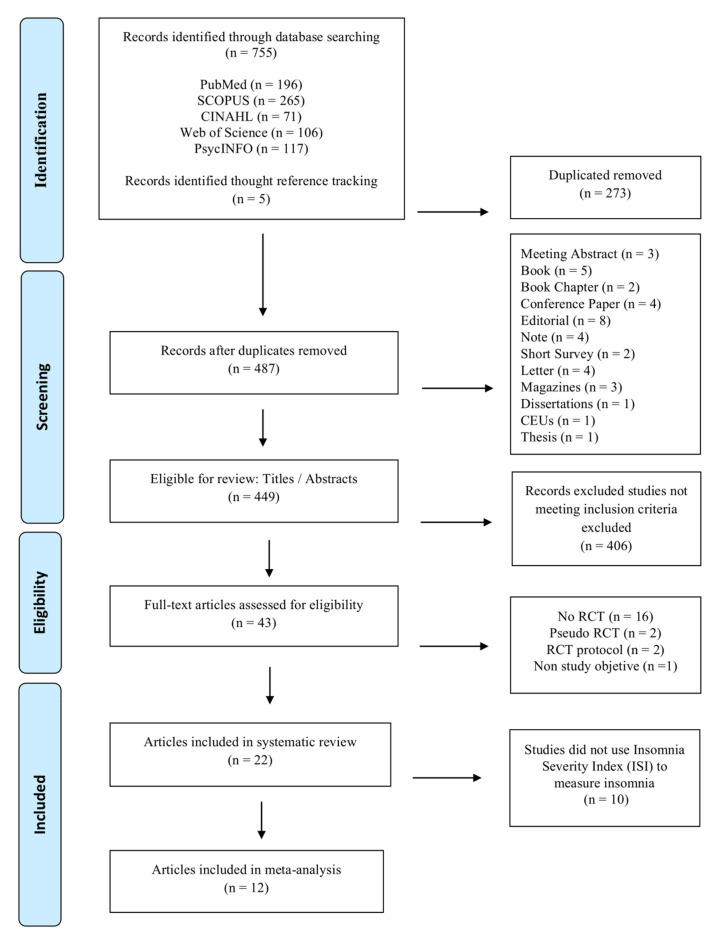
Flowchart of study selection.

Two authors (J.V.E. and R.D.C.) separately examined the 760 records obtained from the internet searches to delete duplicates, thus obtaining an initial sample of 487 references. After three researchers (J.V.E., R.D.C., and J.M.R.S.) had separately read the titles and abstracts to match the eligibility criteria, 406 studies were rejected, leaving 43 search records to be read in full. Lastly, the same three researchers reviewed the complete text. A fourth researcher (A.M.P.G.) acted as a tiebreaker in case of a discrepancy (10%). The final sample consisted of 22 studies, which were included in the review, 12 of which were included in the meta-analysis (Figure 1). 

### 2.5. Data Extraction

One reviewer (J.V.E.) was in charge of extracting the data, which was subsequently verified by another reviewer (R.D.C.). Once the data had been extracted, a table was drawn up including the author, year, country, sample size, scales, type of intervention, number of sessions, monitoring, compared results and quality of the evidence.

### 2.6. Risk of Bias Assessment

Two authors (J.V.E. and R.D.C.) separately used the Cochrane Collaboration tool to assess the quality of the randomized controlled tests [19] with the aim of assessing the risk of bias of the selected studies, by classifying the following dimensions: (I) random sequence generation, (II) allocation concealment, (III) blinding of participants and personnel, (IV) blinding of outcome, (V) incomplete outcome data, (VI) selective reporting (reporting bias) and (VII) other biases. The studies were evaluated in terms of high risk of bias, low risk of bias, or insufficient information, when a criterion could not be evaluated. The kappa coefficient was calculated to measure inter-rater reliability, with a value between 0.92 and 1.

### 2.7. Quality Assessment

The methodological quality assessment was carried out using the CONSORT guidelines for critically assessing randomized clinical trials [20] (Table 1). The studies included were assessed separately by two reviewers (J.V.E. and S.B.T.). Any discrepancy (14%) was resolved by a third reviewer (R.D.C.). Next, the quality of evidence was also assessed using the Grading of Recommendations Assessment Development and Evaluation (GRADE) tool [21], which classified the quality of the evidence as very low, low, moderate, or high, through the risk of bias assessment, imprecision, direction of bias, inconsistency, and suspected publication bias. Also, the kappa coefficient was calculated to measure inter-rater reliability, with a value between 0.88 and 1.

### 2.8. Statistical Analysis

For the meta-analysis, three authors (J.V.E., S.B.T., and M.R.S.) used the Cochrane Review Manager software (RevMan 5.3) to carry out the statistical calculation and create forest plots and funnel plots figures. In addition, the GRADEpro tool was used to create the evidence profile table. The difference between means with a confidence interval of 95% was used to assess the effect of the interventions on work-related insomnia. The heterogeneity was evaluated through the chi-squared test and the I^2^. According to the Cochrane Collaboration tool, heterogeneity is classified as non-significant (0–40%), moderate (30–60%), substantial (50–90%), and considerate (75–100%) [22]. In the studies where there was more than one intervention group, the data were extracted and used as different analyses (e.g., Järnefelt et al., 2019.a—control group sleep hygiene education versus group—based CBT-I; Järnefelt et al., 2019.b—control group sleep hygiene education versus self-help-based CBT-I). For the quantitative analysis, a meta-analysis was carried out together with the pooled effect measure using the Mantel-Haenszel random effects method with a confidence interval of 95% [23]. The assessment of the risk of publication bias was carried out using the funnel plot that represents the effect size on the *X*-axis and the standard error on the *Y*-axis for each included study. At the top of the graph are the largest and most accurate studies, and as we go down, the precision of the studies decreases and they are shifted to the sides by random error. When there is publication bias, this displacement is asymmetric. A sensitivity analysis was carried out for the results of the meta-analysis, in which more than two studies were included in order to ascertain the effect of each of the trials on the results obtained. In addition, a subgroup analysis was performed by considering the insomnia assessment method.

## 3. Results

### 3.1. Characteristics of the Studies

The selected studies are shown in Table 1. Half of the interventions were carried out in the geographical area of Europe, followed by 27.2% in the United States and 22.8% in Asia. It was found that 90.9% of the studies had been published in the last 10 years and 68.1% in the last 5 years, and English was the language used in all of the publications. 

The total sample included 4905 healthy workers (did not have either mental or physical disorder, which interfered with sleep) belonging to different professional sectors such as that of education, communications, health, or the armed forces, being 564 shift workers (11.5%). The average age of the participants was 44 years (SD 5.4). The sample was mostly female (57.5%), with a high level of education (71.9%).

Regarding the design, half of the RCTs, used a wait list control group, 9.1% assessed more than one intervention group, 9.1% had a low number of subjects (since these were pilot studies), 9.1% carried out a cluster randomization, and only one of the studies was multicenter.

Likewise, Table 1 shows the scores obtained in the RCT checklist (CONSORT Statement), taken from the studies included whose mean value was 20.6 (SD = 2.05). It was noted that none reached the maximum score of 25, which is one of the main reasons that most of these studies do not enable the use of double-blind methods and do not provide sufficient information the methods used to generate the random allocation sequence and the likelihood of bias in group assignment. The highest-scoring studies at 23.5 points [24,25]. The third-party blind method, which means that the examiner that has no idea of the patient’s allocation, was present in only one study [26]. In addition, following the last step of MARS recommendation, effects size estimates, including measures of uncertainty, are indicated. 

### 3.2. Intervention Protocols

Of the interventions involved in this study, the majority produced positive results [24,25,26,27,28,29,30,31,32,33,34,36,38,39,40,41,42,44] (*n* = 18) or neutral results [35,37,43] (*n* = 3) with only one exception, which produced negative results [45].

As regards the instrument, twelve studies [24,25,30,31,32,33,34,35,38,41,43,45] used the *Insomnia Severity Index* (ISI) scale in order to measure insomnia. The ISI scale is a tool made up of 7 items that measure the severity of sleep discordance (difficulty falling sleep, difficulty staying asleep, waking up too early in the morning, satisfaction with current sleep pattern, sleep problem interfering with daily functioning, other people’s perception of the apparent deterioration in one’s quality of life and the degree of concern about difficulties in sleeping). Each item is rated on a scale from 0 to 4 and the total score varies from 0 to 28. Depending on the total score, people are classified, according to the severity of their insomnia, in no clinically significant insomnia (0 to 7), subthreshold insomnia (8 to 14), clinical insomnia—moderate severity (15 to 21), and clinical insomnia—severe (22 to 28). The internal consistency for this scale is excellent (α = 0.90) and convergent validity is good. Moreover, an ISI psychometry study revealed that a cut-off score of 10 is acceptable for diagnosing insomnia in community samples. The *Pittsburgh Sleep Quality Index* (PSQI) questionnaire was used in 8 studies [32,33,36,37,39,40,42,44] and a minority of the studies used *Ad Hoc* [26,28] questionnaires or other scales such as the *Athens Insomnia Scale* (AIS) [36] or *The Basic Nordic Sleep Questionnaire* (BSNQ) [29] to measure the level of insomnia. The results obtained after using these tools were finalized and contrasted with objective measurements such as actigraphy. 

Of the 22 studies selected, 68.1% were interventions based on cognitive behavioral therapy (CBT) [24,25,27,30,31,33,34,35,36,39,41,43,44,45], 13.6% on health programs [32,37,40], 9.1% on mindfulness-based therapies [28,42] or expressive writing [38], and only one intervention studied the effect of a drug [26]. The majority of the interventions were carried out through internet-based sessions and the average length of the sessions was around 80 minutes. It is important to note that the specialists who directed and carried out the interventions based on CBT had previously undergone specific training in this type of therapy and in steps to ensure sleep hygiene. Of the studies, 95.4% monitored the results obtained after the intervention, with a follow up ranging from one to twelve months.

#### 3.2.1. Cognitive Behavioral Therapy

CBT-based interventions were the main method in the selected studies, which were based on stimulus control, sleep restriction, and guidelines for sleep hygiene or relaxation. Two different approaches were identified: using interactive internet-based programs on a PC or using a smartphone with added support from SMS or emails [24,25,27,30,31,34,41,44], both of which included goal setting, planning of activities, interactive exercises, audiovisual material, or, in some cases, either face-to-face sessions with a therapist or online sessions with a virtual therapist [29,35,36,39,43,45]. The sessions were conducted either individually or in groups, and were run by psychologists or nurses, with the aim of developing steps to program sleep, control sleep debt, promote sleep, improve lifestyles, decrease stress, control stimuli, and provide training in relaxation.

CBT-I was effective in treating a persistent lack of sleep [27], reducing the severity of insomnia mediated by the reduction of perseverative cognitions and sleep effort [30], and improving the overall quality of the workers’ sleep [44]. Likewise, among the work-related consequences, slight improvements were evident in productivity and presenteeism levels after CBT-I, but not in absenteeism rates, which did not produce statistically significant differences [24,27]. In addition, a moderating effect of burnout was observed on insomnia symptoms [43].

#### 3.2.2. Health Programs

Three studies in particular based their intervention on developing a health program as a way of reducing insomnia levels in workers. Their approaches varied, ranging from the implementation of a physical activity program in the workplace [32] to steps to promote family reconciliation [37,40]. All the programs were conducted in the workplace itself, with voluntary participation and, in two cases, financial incentives.

The results obtained reported increased quality of sleep [32], an increase of one hour per week in the total sleep time, and an improvement in workers’ perceptions regarding the insufficiency of sleep [40]. Likewise, it was noted [37] that these improvements in sleep were statistically more significant among younger workers and in jobs which required a longer physical presence in the workplace.

#### 3.2.3. Mindfulness-Based Therapies and Other Therapies

As regards relaxation and self-help techniques to improve the quantity and quality of sleep, two studies have examined the effects of a mindfulness-based intervention in the workplace [28,42], and another study assessed the effect of an intervention based on expressive writing [38]. The results obtained were positive both in the reduction of insomnia symptoms, with less difficulty in falling asleep at night, and in an increase in the nighttime sleeping hours during the week, and also reported increased levels of sleep quality. 

Finally, only one study [26] evaluated the effects of drug therapy before nighttime sleep: the oral intake of 5 mg of melatonin compared to placebo decreased latency at sleep onset and also increased sleep quality indices.

### 3.3. Risk of Bias in the Studies Included

The assessment of the risk of bias for each study showed that most studies obtained a moderate risk of bias. (Supplementary Materials Figure S2 and Figure S3). Several studies obtained a high risk of bias in one of the dimensions assessed [24,27,28,29,31,33,35,37,39,41,43]. Additionally, 36.3% had an unclear risk of selection bias due to them not providing enough information regarding the randomization process. Of the studies, 77.2% showed bias regarding the blinding of participants and personnel due to the use of the waiting list as a control group or not giving information to confirm it. However, this kind of bias is a common characteristic in studies based on psychotherapeutic self-help interventions [15]. Lastly, 59% of the studies obtained an unclear risk of bias regarding other sources of bias as not enough information was provided for them to be assessed.

The GRADE assessment results indicated that the average quality of the study was acceptable. Moreover, a moderate certainty of evidence was observed for the results.

### 3.4. Intervention Effects

**Figure 2:**
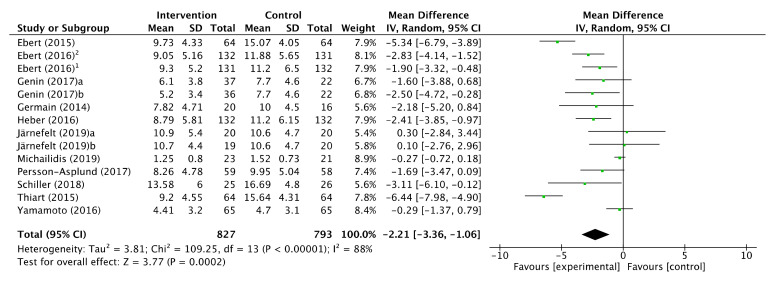
Forest plot of effectiveness of workplace interventions for insomnia symptoms reduction in accordance with Insomnia Severity Index.

**Figure 3:**
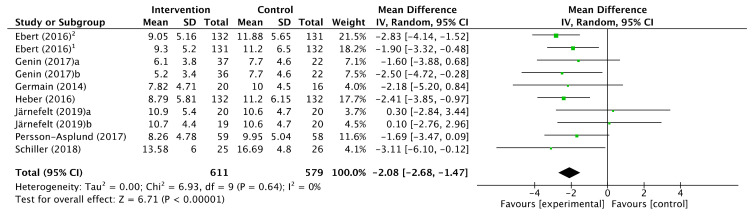
Forest plot of the effectiveness of workplace interventions for the reduction of insomnia symptoms according to the Insomnia Severity Index after removing studies for their outliers.

A total of 12 studies with 14 intervention groups, providing a total sample of 827 workers, informed of an improvement in the scoring on the Insomnia Severity Index as an outcome measure. According to the estimated means difference (MD) of the studies, a moderate reduction in insomnia symptoms was obtained after the intervention (MD −2.21, CI 95%: [−3.36, −1.06]) although the degree of heterogeneity among the studies was assessed as considerable (*p* < 0.00001; I^2^ = 88%). After ruling out 4 studies due to outliers, the effect continued to be moderate (MD −2.08, CI 95%: [−2.68, −1.47]) and the heterogeneity was reduced until it was classified as non-significant (*p* = 0.64); I^2^ = 0%. ( Figure 2; Figure 3).

The exclusion of the studies for their outliers was due to the sensitivity analysis carried out to assess the stability of the pooled estimate with respect to each study individually in the meta-analysis. The studies by Michaidilis (2019), Thiart (2015), Ebert (2015), and Yamamoto (2016) were those which most affected the heterogeneity of the meta-analysis, with I^2^ results of 81, 81, 85, and 89%, respectively.

### 3.5. Funnel Plot

**Figure 4:**
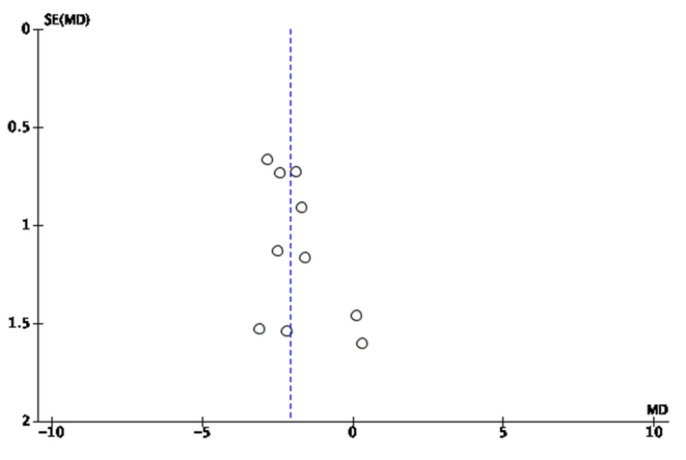
Funnel plot of comparison: insomnia intervention versus control condition.

The funnel plot for the results of the improvement in insomnia symptoms according to the Insomnia Severity Index after the intervention was asymmetric, having had to assess a potential overestimation of the actual effect of the meta-analysis result due to a lack of published studies, although the publication bias assessment may not be completely accurate owing to the small number of studies included (Figure 4).

## 4. Discussion

This systematic review and meta-analysis aimed to identify and evaluate interventions for the reduction of insomnia as a risk factor in occupational health. Twenty-two studies were chosen for the systematic review and all met the inclusion criteria. The findings showed that the majority of the interventions were based on cognitive behavioral therapy, established on an individual or group basis and carried out via face-to-face or via internet. A moderate positive effect was found for the reduction in insomnia after the intervention, with a non-significant heterogeneity, after ruling out 4 studies for their atypical values. Twelve studies were selected to perform the meta-analysis, which formed a total of 14 intervention groups and whose common denominator was the use of the *Insomnia Severity Index* (ISI) as an outcome measure to evaluate the efficiency of the interventions on insomnia.

CBT in the workplace has demonstrated its effectiveness with other illnesses; a recent meta-analysis of interventions that measured symptoms of depression noted that CBT reduced the level of these symptoms among workers [46]. Another meta-analysis showed that resilience interventions based on CBT (with a combination of mindfulness techniques) had a positive impact on individual resilience [47]. Nevertheless, in the context of the psychological well-being of employees and increasing work effectiveness, another previous meta-analysis noted no statistically significant differences in outcomes between studies using CBT therapy compared with other psychological approaches, although it pointed out that mental health interventions improved the workers’ psychological well-being and increased work effectiveness [48].

However, CBT focusing on mental illnesses in a non-working-population concluded that the use of Digital Health Interventions (DHIs) had proved effective. Another meta-analysis in children and adolescents showed a benefit in using CBT-based technology (internet and computers) to reduce depression and anxiety [49]. As for the use of smartphones in mental health interventions, another meta-analysis pointed out that psychological interventions delivered via these devices reduced anxiety [50]. Nevertheless, a recent meta-analysis has showed that such interventions with smartphones were characterized by high rates of attrition and low adherence [51].

All the researchers included in this study directly or indirectly investigated insomnia in their interventions. When insomnia was approached indirectly, it was through interventions whose primary objective was to evaluate the effectiveness of interventions focused on reducing levels of work-related stress, rumination, anxiety, fatigue, and depression. In our review, the majority of the interventions reported positive results. This coincides with what has been shown by other meta-analyses, which revealed positive and efficient effects in the general population for cognitive behavioral therapy on insomnia [52,53], which was the predominant treatment option in the studies included in our meta-analysis. Each of these studies had their own particular characteristics, but in general, all of them demonstrated a sufficient degree of methodological reliability and quality regarding the interventions on work-related insomnia.

Regarding shift work, several studies included in this systematic review did not observe the variable shift work as an influencing factor in the appearance of insomnia. However, a recent review of studies that has shown a direct relationship between shift work (regardless of its characteristics and context) and the appearance of insomnia in the working population [54].

To our knowledge, this is the first time that a research team has described and discussed in detail the methodology used in the clinical trials selected, whose specific aims focused on assessing the impact of interventions for work-related insomnia and/or other aspects indirectly linked to or acting as precursors of insomnia in occupational health. 

In accordance with the provisions of the PRISMA Statement, randomized sequence generation is an extremely important factor, as this process can heavily influence the results [17]. In our study, the Cochrane Collaboration tool was used to evaluate, among other things, this randomization process in terms of its implementation and the way it is described in the scientific articles. One third of the studies included did not provide enough information to allow us to assess this risk of bias, which made a big difference to the research results. However, the absence of a description of the process does not necessarily imply that it was not carried out, although it does make it difficult to identify. 

Furthermore, in accordance with the amended CONSORT guidelines, for interventions with non-pharmacological approaches, studies that do not include a double-blinding are not validated, although this option is considered in cases where the characteristics of the sample or the intervention itself do not allow it [55]. Such is the case of the interventions included in this meta-analysis, in which half of the studies used a waiting list as a control group. This condition in the design of the interventions involves a high risk of blinding bias and can cause a lower output in the estimates of the results obtained. Similarly, it may lead to a lack of involvement of the control group members, as well as higher levels of frustration during the waiting period. 

Regarding clinical significance and clinical relevance of the results reported by this study, this meta-analysis has shown that the intervention is useful to improve occupational insomnia since they reduced an average of 2.08 points the values of the Insomnia Severity Index scale (ISI) of the intervention group versus the control group. Specifically, the alpha error obtained (*p*-value) was less than 0.1%. Furthermore, the confidence interval for this difference in means is very narrow, ranging from −3.36 to −1.06, which shows the high precision (low sample error of the study).

On the other hand, we know that any difference between the two groups being compared, no matter how small, can become “statistically significant” if the sample size increases considerably. However, this is not the case in our meta-analysis, where the sample size is not excessively large (611 and 579 workers, for intervention and control, respectively). As can be calculated from the data shown in Figure 3, the workers in the control group obtained a mean score of 11.15 points in the ISI scale. The meta-analysis reported that the therapeutic intervention produced a decrease of 2.08 points on average. An average reduction of 2.08 on an average score of 11.15 corresponds to a reduction of 19.6%. In other words, the therapeutic intervention reduces the insomnia score by around 20%. With which we consider that the clinical relevance is moderate.

The analysis of these methodological issues reveals aspects to be taken into account in future studies that address insomnia in the workforce. In line with the natural difficulties already addressed in this line of research, other relevant aspects for minimizing bias are important and easily applicable. Attempts to solve these methodological problems in studies that address insomnia may be key to improving the quality of research in this field.

This study has certain limitations regarding the systematic review and meta-analysis. Although the search included 5 databases, it is likely that other studies indexed in other databases not previously consulted have not been included. The author’s bias must also be noted as a limitation, due to the fact that various studies were written by the same research team. Another limitation was the exclusive choice of the values obtained on the ISI scale as an outcome or effect measure for evaluating the effectiveness of interventions, as this questionnaire that measures the level and severity of insomnia was the most widely used in the search records included in our study. In some studies, the ages of the participants were not recorded, and this information is significant because there are studies that have shown the influence of age on the effect size for different sleep variables depending on the intervention [56]. Lastly, the professional sector to which the participants belonged in the majority of studies included in the meta-analysis required a high educational level, which causes a lower generalization of the results for other professional sectors with fewer educational requirements.

## 5. Conclusions

This systematic review and meta-analysis analyses the evidence provided by RCTs to improve or reduce insomnia in the workforce, finding a moderate positive effect after the intervention (MD −2.08, CI 95%: [−2.68, −1.47]) when using the ISI scale as a reference. The majority of the interventions included in this study use cognitive behavioral therapy either face-to-face or via Internet, followed by health programs, meditation techniques, self-help, and pharmacology as tools for combating insomnia and raising the worker’s awareness of the causes and effects of insomnia: all of which, with the exception of one, showed positive results in meeting their aims. We can therefore conclude that interventions for insomnia in the workplace are effective. As regards work, although there were improvements in the productivity, presenteeism, and job burnout indices, none of the studies reported any results for insomnia with this variable, or with accidents in the workplace, despite the fact that some studies included ‘being unemployed’ as a study variable. As for sleeping health, almost all the interventions noted improvements in the quality of sleep and a decrease in insomnia symptoms due to increased weekly hours of sleep and a reduction in the latency at sleep onset, which led to an improvement in the workers’ perception of sleep satisfaction.

Up to now, few interventions with results have been carried out in insomnia in workers, despite the numerous benefits obtained after carrying out highly effective measures to reduce stress and improve family reconciliation. It is up to organizations, administrations, and employers to continue working and investing in labor policies aimed at improving workers’ sleep, in order to ensure greater productivity and job security.

## Figures and Tables

**Table 1:** Characteristic of studies included in the systematic review and meta-analysis.

Study	Country	Participants	N	Scale (Insomnia)	Type Intervention	Nº Sessions; Duration Sessions (Min)	Follow-Up	Effect Sizes (IG-CG)	Methodological Quality (CONSORT)
Bostock et al., 2016 [27]	UK	Office-based staff	270	SCI	dCBT	6/-	8 weeks/3 months	1.20 [−0.82, 3.22]	18/25
Crain et al., 2017 [28]	USA	Teachers	113	Ad Hoc questionnaire	WMT	11/120–420	up to 3 months	−0.40 [−0.99. 0,19]	18/25
Dalgaard et al., 2014 [29]	Denmark	Workers on sick leave	137	BNSQ	CBT	6/60 1–2/60–120	4, 10 months	−1.44 [−3.03, −0.15]	21/25
Ebert et al., 2015 [30]	USA	Teachers	128	ISI	GET.ON recovery (CBT)	6/45–60	8 weeks/6 months	−5.34 [−6.79, −3.89]	22/25
Ebert, Lehr et al., 2016 [24]	USA	General working population	264	ISI	GET-ON stress (iSIM)	8/45–60	7 weeks/ 6 months	−2.83 [−4.14, −1.52]	23,5/25
Ebert, Heber et al., 2016 [31]	USA	General working population	264	ISI	GET-ON stress (iSIM)	8/45–60	7 weeks/6 months	−1.90 [−3.32, −0.40]	21/25
Genin et al., 2017 [32]	France	Office employees	95	ISI	Worksite physical activity program	40/45	5 months	−1.60 [−3.88, 0.68] ^a^ −2.50 [−4.72, −0.28] ^b^	17/25
Germain et al., 2014 [33]	USA	Military	40	ISI, PSQI	BBTI-MV	2 + 2/20–45	1, 6 months	−2.18 [−5.20, 0.84] 3.25 [2.22, 4.29]	19,5/25
Heber ar al., 2016 [34]	Germany	General working population	264	ISI	GET.ON stress (iSMI)	7 + 1/30	7 weeks/6 months	−2.41 [−3.85, −0.97]	23/25
Järnefelt et al., 2019 [35]	Finland	Shift workers	83	ISI	gCBT-I/sCBT-I	(6/90) (6/45)	6 months	0.30 [−2.84, 3.44] ^a^ 0.10 [−2.76, 2.96] ^b^	22/25
Kaku et al., 2012 [36]	Japan	Design engineers	223	PSQI	CBT + SH	20/30	3 months	1.9 [0.6, 3.4]	21/25
Marino et al., 2016 [37]	USA	Employees and managers (nursing homes)	1522/184	PSQI	STAR	4 + 3/60	6, 12 months	0.00 [−0.10, 0.10] ^a^ −0.14 [−0.43, 0.15] ^b^	20/25
Michailidis & Cropley, 2019 [38]	UK	General working population	44	ISI	Expressive writing	3/20	1, 3 months	−0.27 [−0.72, 0.18]	22,5/25
Nishinoue et al., 2012 [39]	Japan	White-collar employees	127	PSQI	CBT + SH	1/30	3 months	1.0 [0.02, 2.0]	18,5/25
Olson et al., 2015 [40]	USA	Employees (information technology)	474	PSQI	STAR	3/480	6, 12 months	−0.10 [−0.32, 0.12]	23/25
Persson Asplund et al., 2 018 [41]	Sweden	Managers	117	ISI	iSIM	8/120–180	6 months	−1.69 [3.47, 0.09]	23/25
Querstret et al., 2017 [42]	UK	General working population	118	PSQI	Mindfullness (online)	10/30 + 10	3, 6 months	−0.41 [−0.80, −0.02]	20,5/25
Sadeghniiat-Haghighi et al., 2008 [26]	Iran	Nurses	86	Ad Hoc questionnaire	Pharmacological	3/-	none	−0.11 [−0.29. 0.07]	18,5/25
Schiller et al., 2018 [43]	Sweden	General working population	51	ISI	CBT	5/120	3 months	−3.11 [−6.10, −0.12]	21/25
Suzuki et al., 2008 [44]	Japan	General working population	43	CSQI/PSQI	CBT	2 weeks	3 weeks	−2.09 [−6.70, 2.52] 1.18 [−1.20, 3.56]	17,5/25
Thiart et al., 2015 [25]	Germany	Teachers	128	ISI	GET.ON Recovery (CBT)	6/-	6 months	−6.44 [−7.98, −4.90]	23,5/25
Yamamoto et al., 2016 [45]	Japan	Office workers	130	ISI	CBT-I	2/30 + 60	3 months	−0.29 [−1.37, 0.79]	21/25

Abbreviations: N, total simple size; IG, intervention group; CG, control group; SCI, sleep condition indicator; dCBT, digital cognitive behavioral therapy; WMT, workplace mindfulness training; BNSQ, basic nordic sleep questionnaire; CBT, cognitive–behavioral therapy; ISI, insomnia severity index; iSMI, internet-based stress management intervention; ^a^, intervention group 1; ^b^, intervention group 2; PSQI, pittsburgh sleep quality index; BBTI-MV, military version of a brief behavioral treatment of insomnia; gCBT-I, group-based CBT-I; sCBT-I; self-help-based CBT-I; SH, sleep hygiene; STAR; support. transform, achieve. results; CSQI, current sleep quality index; CBT-I, cognitive behavioral therapy of insomnia.

## Supplementary Materials

The following are available online at https://www.mdpi.com/1660-4601/17/17/6401/s1, Table S1. Search strategy, Figure S2. Risk of bias graph: review authors’ judgements about each risk of bias item presented as percentages across all included studies, Figure S3. Risk of bias summary: review authors’ judgements about each risk of bias item for each included study.

## References

[1] Baglioni C., Altena E., Bjorvatn B., Blom K., Bothelius K., Devoto A., Espie C.A., Frase L., Gavriloff D., Tuuliki H. (2019). The European Academy for Cognitive Behavioural Therapy for Insomnia: An initiative of the European Insomnia Network to promote implementation and dissemination of treatment. J. Sleep Res..

[2] American Psychiatric Association (2016). Actualización del DSM-5^®^.

[3] Buysse D.J. (2013). Insomnia. JAMA.

[4] Riemann D., Baglioni C., Bassetti C., Bjorvatn B., Dolenc Groselj L., Ellis J.G., Espie C.A., Garcia-Borreguero D., Gjerstad M., Gonçalves M. (2017). European guideline for the diagnosis and treatment of insomnia. J. Sleep Res..

[5] Bjorvatn B., Meland E., Flo E., Mildestvedt T. (2017). High prevalence of insomnia and hypnotic use in patients visiting their general practitioner. Fam. Pract..

[6] Yang B., Wang Y., Cui F., Huang T., Sheng P., Shi T., Huang C., Lan Y., Huang Y.-N. (2018). Association between insomnia and job stress: A meta-analysis. Sleep Breath..

[7] Rajaratnam S.M.W., Licamele L., Birznieks G. (2015). Delayed sleep phase disorder risk is associated with absenteeism and impaired functioning. Sleep Health.

[8] Schlack R., Hapke U., Maske U., Busch M., Cohrs S. (2013). Häufigkeit und Verteilung von Schlafproblemen und Insomnie in der deutschen Erwachsenenbevölkerung. Bundesgesundheitsblatt—Gesundheitsforsch—Gesundheitsschutz.

[9] DiBonaventura M., Richard L., Kumar M., Forsythe A., Flores N.M., Moline M. (2015). The association between insomnia and insomnia treatment side effects on health status, work productivity, and healthcare resource use. PLoS ONE.

[10] Hu C.-J., Hong R.-M., Yeh G.-L., Hsieh I.-C. (2019). Insomnia, Work-Related Burnout, and Eating Habits Affecting the Work Ability of Flight Attendants. Aerosp. Med. Hum. Perform..

[11] Laugsand L.E., Strand L.B., Vatten L.J., Janszky I., Bjørngaard J.H. (2014). Insomnia Symptoms and Risk for Unintentional Fatal Injuries—The HUNT Study. Sleep.

[12] Sivertsen B., Overland S., Bjorvatn B., Maeland J.G., Mykletun A. (2009). Does insomnia predict sick leave? The Hordaland Health Study. J. Psychosom. Res..

[13] Blafoss R., Sundstrup E., Jakobsen M.D., Bay H., Garde A.H., Andersen L.L. (2019). Are Insomnia Type Sleep Problems Associated With a Less Physically Active Lifestyle? A Cross-Sectional Study Among 7,700 Adults From the General Working Population. Front. Public Health.

[14] Garbarino S., Magnavita N. (2019). Sleep problems are a strong predictor of stress-related metabolic changes in police officers. A prospective study. PLoS ONE.

[15] Phillips E.A., Gordeev V.S., Schreyögg J. (2019). Effectiveness of occupational e-mental health interventions: A systematic review and meta-analysis of randomized controlled trials. Scand. J. Work Environ. Health.

[16] Lehr D., Heber E., Sieland B., Hillert A., Funk B., Ebert D.D. (2016). “Occupational eMental Health” in der Lehrergesundheit: Ein metaanalytisches Review zur Wirksamkeit von Online-Gesundheitstrainings bei Lehrkräften. Pravention und Gesundheitsforderung.

[17] Moher D., Shamseer L., Clarke M., Ghersi D., Liberati A., Petticrew M., Shekelle P., Stewart L.A. (2015). PRISMA-P Group Preferred reporting items for systematic review and meta-analysis protocols (PRISMA-P) 2015 statement. Syst. Rev..

[18] Cooper H. (2017). Research Synthesis and Meta-analysis: A Step-by-Step Approach.

[19] Higgins J.P.T., Altman D.G., Gøtzsche P.C., Jüni P., Moher D., Oxman A.D., Savović J., Schulz K.F., Weeks L., Sterne J.A.C. (2011). The Cochrane Collaboration’s tool for assessing risk of bias in randomised trials. BMJ.

[20] Cobos-Carbó A., Augustovski F. (2011). Declaración CONSORT 2010: Actualización de la lista de comprobación para informar ensayos clínicos aleatorizados de grupos paralelos. Med. Clin..

[21] Jaeschke R., Guyatt G.H., Dellinger P., Schunemann H., Levy M.M., Kunz R., Norris S., Bion J. (2008). Use of GRADE grid to reach decisions on clinical practice guidelines when consensus is elusive. BMJ.

[22] Higgins J.P.T., Thomas J. (2019). Cochrane Handbook for Systematic Reviews of Interventions.

[23] DerSimonian R., Laird N. (1986). Meta-analysis in clinical trials. Contemp. Clin. Trials.

[24] Ebert D.D., Lehr D., Heber E., Riper H., Cuijpers P., Berking M. (2016). Internet- and mobile-based stress management for employees with adherence-focused guidance: Efficacy and mechanism of change. Scand. J. Work Environ. Health.

[25] Thiart H., Lehr D., Ebert D.D., Berking M., Riper H. (2015). Log in and breathe out: Internet-based recovery training for sleepless employees with work-related strain—Results of a randomized controlled trial. Scand. J. Work Environ. Health.

[26] Sadeghniiat-Haghighi K., Aminian O., Pouryaghoub G., Yazdi Z. (2008). Efficacy and hypnotic effects of melatonin in shift-work nurses: Double-blind, placebo-controlled crossover trial. J. Circadian Rhythms.

[27] Bostock S., Luik A.I., Espie C.A. (2016). Sleep and productivity benefits of digital cognitive behavioral therapy for insomnia a randomized controlled trial conducted in the workplace environment. J. Occup. Environ. Med..

[28] Crain T.L., Schonert-Reichl K.A., Roeser R.W. (2017). Cultivating teacher mindfulness: Effects of a randomized controlled trial on work, home, and sleep outcomes. J. Occup. Health Psychol..

[29] Dalgaard L., Eskildsen A., Carstensen O., Willert M.V., Andersen J.H., Glasscock D.J. (2014). Changes in self-reported sleep and cognitive failures: A randomized controlled trial of a stress management intervention. Scand. J. Work Environ. Health.

[30] Ebert D.D., Berking M., Thiart H., Riper H., Laferton J.A.C., Cuijpers P., Sieland B., Lehr D. (2015). Restoring depleted resources: Efficacy and mechanisms of change of an internet-based unguided recovery training for better sleep and psychological detachment from work. Health Psychol..

[31] Ebert D.D., Heber E., Berking M., Riper H., Cuijpers P., Funk B., Lehr D. (2016). Self-guided internet-based and mobile-based stress management for employees: Results of a randomised controlled trial. Occup. Environ. Med..

[32] Genin P.M., Degoutte F., Finaud J., Pereira B., Thivel D., Duclos M. (2017). Effect of a 5-Month Worksite Physical Activity Program on Tertiary Employees Overall Health and Fitness. J. Occup. Environ. Med..

[33] Germain A., Richardson R., Stocker R., Mammen O., Hall M., Bramoweth A.D., Begley A., Rode N., Frank E., Haas G. (2014). Treatment for Insomnia in Combat-Exposed OEF/OIF/OND Military Veterans: Preliminary Randomized Controlled Trial. Behav. Res. Ther..

[34] Heber E., Lehr D., Ebert D.D., Berking M., Riper H. (2016). Web-Based and Mobile Stress Management Intervention for Employees: A Randomized Controlled Trial. J. Med. Internet Res..

[35] Heli J., Mikko H., Mikael S., Jussi V., Teemu P., Kari-Pekka M., Christer H. (2019). Cognitive behavioural therapy interventions for insomnia among shift workers: RCT in an occupational health setting. Int. Arch. Occup. Environ. Health.

[36] Kaku A., Nishinoue N., Takano T., Eto R., Kato N., Ono Y. (2012). Randomized controlled trial on the effects of a combined sleep hygiene education and behavioral approach program on sleep quality in workers with insomnia. Ind. Health.

[37] Marino M., Berkman L.F., Olson R., Killerby M., Erickson L., Buxton O.M., Lee S., King R., Moen P., Kossek E.E. (2016). The effects of a cluster randomized controlled workplace intervention on sleep and work-family conflict outcomes in an extended care setting. Sleep Health.

[38] Michailidis E., Cropley M. (2019). Testing the benefits of expressive writing for workplace embitterment: A randomized control trial. Eur. J. Work Organ. Psychol..

[39] Nishinoue N., Takano T., Kaku A., Eto R., Kato N., Ono Y., Tanaka K. (2012). Effects of sleep hygiene education and behavioral therapy on sleep quality of white-collar workers: A randomized controlled trial. Ind. Health.

[40] Olson R., Crain T.L., Bodner T.E., King R., Hammer L.B., Klein L.C., Erickson L., Moen P., Berkman L.F., Buxton O.M. (2015). A workplace intervention improves sleep: Results from the randomized controlled Work, Family, and Health Study. Sleep Health.

[41] Persson Asplund R., Dagoo J., Fjellstrom I., Niemi L., Hansson K., Zeraati F., Ziuzina M., Geraedts A., Ljotsson B., Carlbring P. (2018). Internet-based stress management for distressed managers: Results from a randomised controlled trial. Occup. Environ. Med..

[42] Querstret D., Cropley M., Fife-Schaw C. (2017). Internet-based instructor-led mindfulness for work-related rumination, fatigue, and sleep: Assessing facets of mindfulness as mechanisms of change. A randomized waitlist control trial. J. Occup. Health Psychol..

[43] Schiller H., Söderström M., Lekander M., Rajaleid K., Kecklund G. (2018). A randomized controlled intervention of workplace-based group cognitive behavioral therapy for insomnia. Int. Arch. Occup. Environ. Health.

[44] Suzuki E., Tsuchiya M., Hirokawa K., Taniguchi T., Mitsuhashi T., Kawakami N. (2008). Evaluation of an internet-based self-help program for better quality of sleep among Japanese workers: A randomized controlled trial. J. Occup. Health.

[45] Yamamoto M., Sasaki N., Somemura H., Nakamura S., Kaneita Y., Uchiyama M., Tanaka K. (2016). Efficacy of sleep education program based on principles of cognitive behavioral therapy to alleviate workers’ distress. Sleep Biol. Rhythms.

[46] Nigatu Y.T., Huang J., Rao S., Gillis K., Merali Z., Wang J. (2019). Indicated Prevention Interventions in the Workplace for Depressive Symptoms: A Systematic Review and Meta-analysis. Am. J. Prev. Med..

[47] Joyce S., Shand F., Tighe J., Laurent S.J., Bryant R.A., Harvey S.B. (2018). Road to resilience: A systematic review and meta-analysis of resilience training programmes and interventions. BMJ Open.

[48] Carolan S., Harris P.R., Cavanagh K. (2017). Improving Employee Well-Being and Effectiveness: Systematic Review and Meta-Analysis of Web-Based Psychological Interventions Delivered in the Workplace. J. Med. Internet Res..

[49] Grist R., Croker A., Denne M., Stallard P. (2019). Technology Delivered Interventions for Depression and Anxiety in Children and Adolescents: A Systematic Review and Meta-analysis. Clin. Child Fam. Psychol. Rev..

[50] Firth J., Torous J., Nicholas J., Carney R., Rosenbaum S., Sarris J. (2017). Can smartphone mental health interventions reduce symptoms of anxiety? A meta-analysis of randomized controlled trials. J. Affect. Disord..

[51] Linardon J., Fuller-Tyszkiewicz M. (2020). Attrition and adherence in smartphone-delivered interventions for mental health problems: A systematic and meta-analytic review. J. Consult. Clin. Psychol..

[52] Zachariae R., Lyby M.S., Ritterband L.M., O’Toole M.S. (2016). Efficacy of internet-delivered cognitive-behavioral therapy for insomnia—A systematic review and meta-analysis of randomized controlled trials. Sleep Med. Rev..

[53] Ho F.Y.-Y., Chung K.-F., Yeung W.-F., Ng T.H., Kwan K.-S., Yung K.-P., Cheng S.K. (2015). Self-help cognitive-behavioral therapy for insomnia: A meta-analysis of randomized controlled trials. Sleep Med. Rev..

[54] Vega-Escaño J., Porcel-Gálvez A.M., Barrientos-Trigo S., Romero-Sanchez J.M., de Diego-Cordero R. (2020). Turnicity as a determining factor in the occurrence of insomnia in the working population: A systematic review. Rev. Esp. Salud Publica.

[55] Boutron I., Moher D., Altman D.G., Schulz K.F., Ravaud P. (2008). Extending the CONSORT statement to randomized trials of nonpharmacologic treatment: Explanation and elaboration. Ann. Intern. Med..

[56] Irwin M.R., Cole J.C., Nicassio P.M. (2006). Comparative meta-analysis of behavioral interventions for insomnia and their efficacy in middle-aged adults and in older adults 55+ years of age. Health Psychol..

