# Supplementary material for: Insomnia Interventions in the Workplace: A Systematic Review and Meta-Analysis
Source: Int J Environ Res Public Health. 2020 Sep 2;17(17):6401. doi: 10.3390/ijerph17176401 (PMC7504457; doi:10.3390/ijerph17176401)
Supplement: Supplementary file 1 [file ijerph-17-06401-s001.pdf]

Supplementary Materials:

Table S1. Search strategy

Database	*Search Strategy*
**PubMed**	*((((("sleep initiation and maintenance disorders"[MeSH Terms]) OR ((("sleep"[All Fields] AND "initiation"[All Fields]) AND "maintenance"[All Fields]) AND "disorders"[All Fields])) OR "sleep initiation and maintenance disorders"[All Fields]) OR "insomnia"[All Fields]) OR "insomnias"[All Fields]) AND "occupational health"[All Fields]*
**SCOPUS**	*(TITLE-ABS-KEY (insomnia) AND TITLE-ABS-KEY ("occupational health"))*
**CINAHL**	*(insomnia AND "occupational health")*
**Web of Science**	*TS= (insomnia AND "occupational health")*
PsycINFO	*Insomnia AND "occupational health"*

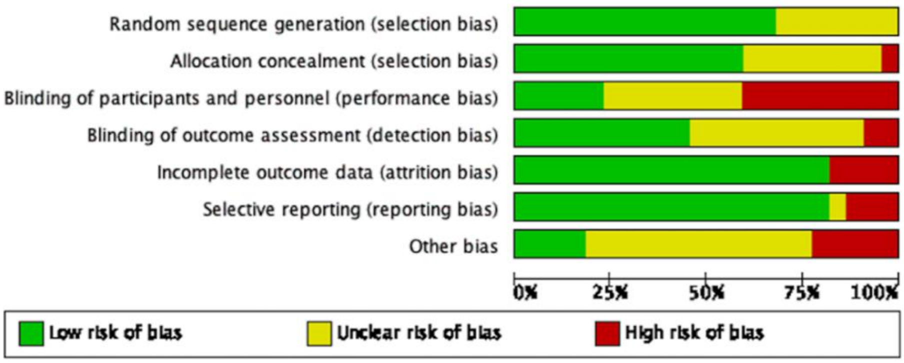

Figure S2. Risk of bias graph: review authors' judgements about each risk of bias item presented as percentages across all included studies.

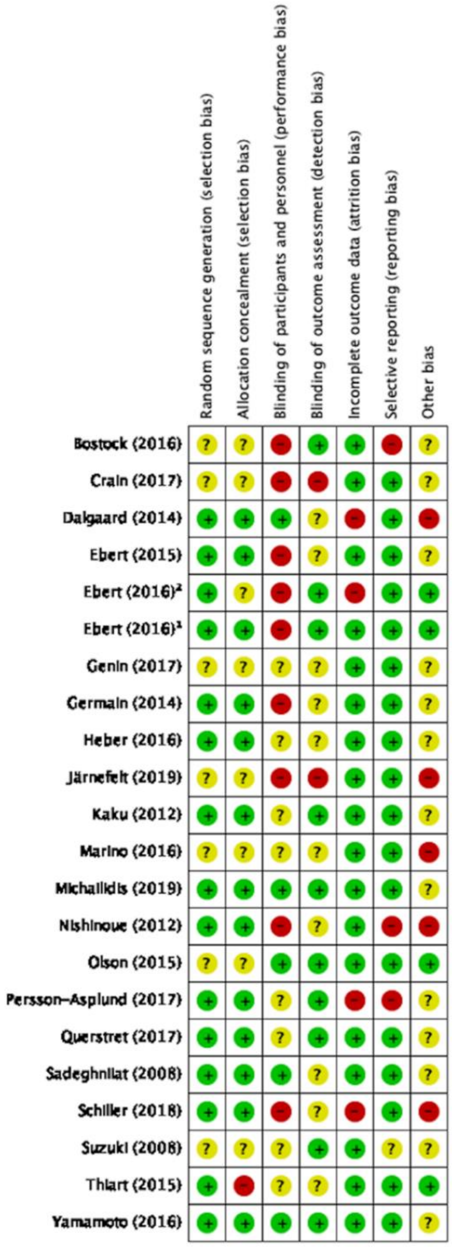

Figure S3. Risk of bias summary: review authors' judgements about each risk of bias item for each included study.
